# Spontaneous Pulmonary Embolism Leading to Sudden Cardiac Arrest and Perimortem C-Section in a 39-Week Parturient During Induction of Labor: A Case Report

**DOI:** 10.7759/cureus.29121

**Published:** 2022-09-13

**Authors:** Cameron Howard, Onassis Naim, Grace Chalhoub, Edwin Rodriguez, Jean Miles

**Affiliations:** 1 Anesthesiology, Memorial Healthcare System, Hollywood, USA; 2 Anesthesiology, Florida International University, Herbert Wertheim College of Medicine, Florida, USA

**Keywords:** case report, labor induction, pulmonary embolism, intrapartum maternal cardiac arrest, perimortem cesarean delivery

## Abstract

We report the successful salvage of mother and baby after a perimortem cesarean delivery (PMCD) complicated by a 21-minute asystolic maternal cardiac arrest (MCA) that was precipitated by a pulmonary embolism during the early stages of induction of labor. With rapid PMCD, recovery of maternal quality of life is possible even after prolonged resuscitation.

## Introduction

Based on an assessment of maternal cardiac arrest (MCA) in the United Kingdom, the incidence is 2.78 per 100,000 maternities (1:36,000) [1]. Amongst patients admitted for delivery in the United States, data shows a higher rate at 1:12,000 with hemorrhage, heart failure, or amniotic fluid embolism (AFE) as the most frequent causes [2]. Parturients who suffer an MCA face a mortality rate between 42% to 58% [1,3], despite basic life support (BLS) and advanced life support (ALS). This is due to the ineffectiveness of chest compressions from aortocaval compression during late pregnancy [4]. The time it takes to start ALS/BLS has less effect on maternal survival than the time from collapse to perimortem cesarean delivery (PMCD) with a median time to PMCD for survivors being three minutes versus 12 minutes for decedents [1].

Seventy-nine percent to 87% of women presenting with MCA and a viable pregnancy are treated with a PMCD [3,5]. PMCD should be considered within four minutes of an MCA with ongoing cardiopulmonary resuscitation (CPR), and with the goal of delivery within five minutes [6]. A survey in Japan found 18 occurrences from 2010-2015; six out of twelve patients were discharged after attempted resuscitations (three with no sequelae, three with deficits, three in a vegetative state, and three deceased) [7]. Out of 18 neonates, three were discharged with no sequelae [7]. The time from MCA to PMCD was three minutes in survivors versus 22 and 36 minutes in deceased and encephalopathic neonates [7]. In a Netherlands study of PMCD, only two out of 11 parturients survived, both of whom were in the hospital at the time of arrest [3]. A recent study of PMCD found that four out of 57 cases were below the four-minute limit with an average of 16.6 minutes [5]. Despite the average delay in PMCD, 60.6% achieved a return of spontaneous circulation (ROSC) and 54% survived to discharge [5]. Neonatal survival was reported at 63.6% [5]. The patient has given written Health Insurance Portability and Accountability Act authorization for publication.

## Case presentation

We report the case of a 36-year-old G2P1 parturient with a past medical history of asthma admitted at 39 5/7 weeks for induction of labor. She was admitted to a labor and delivery suite. The patient received oral misoprostol 25 mg, intravenous nalbuphine 20 mg, and intravenous vancomycin 1000 mg. Labor epidural anesthesia was not requested by the patient. Six hours after misoprostol administration, with no rupture of membranes (ROM), she experienced a painful contraction followed by acute shortness of breath followed immediately by loss of consciousness. An obstetric rescue alert was called concurrent with the Society of Obstetric Anesthesia & Perinatology (SOAP) guidelines [8]. Her presenting rhythm was asystole. On initial collapse, the working diagnosis was a seizure and postictal state. Compressions were started immediately and a PMCD incision was made four minutes later in the labor and delivery suite. The procedure began at the recommended four-minute mark, but delivery occurred six minutes post-MCA which falls just short of the five-minute goal [6]. The five-minute delivery goal may be unrealistic as data places the average time from MCA to PMCD at 10 minutes +/- 7.2 minutes [5]. Collaboration between nursing, anesthesia, obstetric, surgical, intensivist, and interventional radiology teams led to the successful delivery of a healthy infant six minutes after the start of compressions (Appearance, pulse, grimace, activity, and respiration (APGAR) score was 6 at the first minute and 8 at five minutes). Placental abruption was noted. 

After delivery, the mother continued with asystole with agonal beats. Advanced cardiovascular life support (ACLS) protocol was initiated with a successful ROSC after 21 minutes. The mother was transferred to the operating room where her abdomen was further explored. A central line and arterial line were placed, and she was started on an epinephrine infusion at a rate of 10 mcg/min for shock. On exploration, she was found to have an expanding left adnexal hematoma. Massive transfusion protocol was initiated for blood loss and disseminated intravascular coagulation (DIC). Initial international normalized ratio, partial thromboplastin time, and fibrinogen were 2.2, 136.6 seconds, and <50 mg/dL, respectively. Vascular surgery was consulted, and the team excised the left adnexa to control the hemorrhage. 

The patient was transferred to the intensive care unit where she continued to require ventilatory and circulatory support including blood transfusion, infusions of epinephrine and vasopressin as well as inhaled nitric oxide in order to lower pulmonary artery pressure and support the right ventricle. Bedside echocardiogram showed a dilated right ventricle, McConnell's sign (right ventricular free wall akinesia with sparing of the apex), and high suspicion of a clot in the right ventricle. A CT scan was obtained which showed right lower lobe segmental and sub-segmental pulmonary emboli. She was taken to interventional radiology where a uterine artery embolization was performed, and a right-sided pulmonary artery catheter was placed for tissue plasminogen activator infusion.

**Figure 1:**
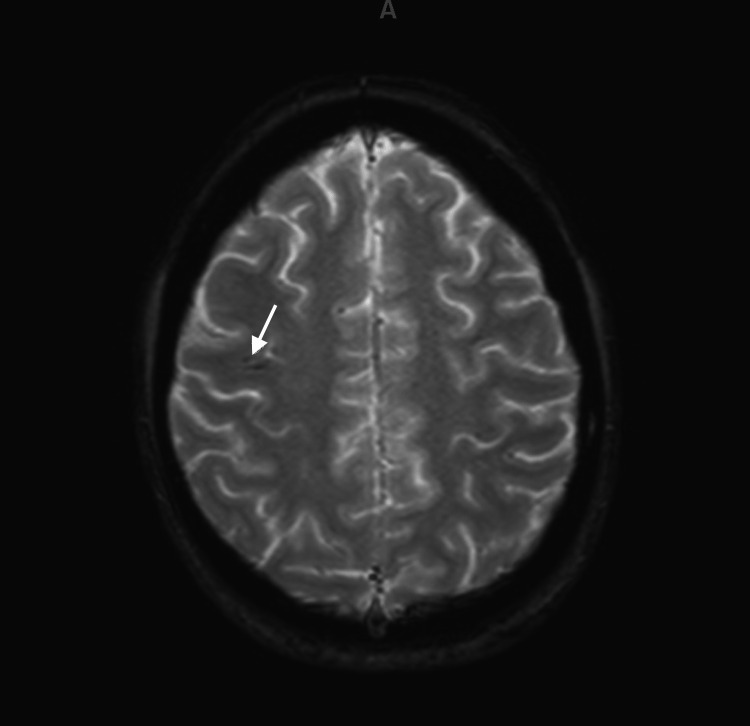
Brain MRI showing chronic cerebral hemispheric watershed infarct

The patient was then transferred to a quaternary facility for potential extracorporeal membrane oxygenation which was later not deemed necessary. DIC was essentially corrected by 36 hours. CT of the brain found subtle hypoattenuation along the frontal lobe. She was extubated on postpartum day 4. She was seen by neurology and psychiatry and subsequently diagnosed with Abulia. Follow-up MRI of the brain showed chronic cerebral hemispheric watershed infarcts with chronic associated microhemorrhage as well as chronic cerebellar infarcts (Figure 1).

Despite these radiologic findings, she improved clinically and was discharged on day 18 for rehabilitation, then home on day 25 with ongoing physical, occupational, and speech therapy. The infant was previously discharged on postpartum day 3 with no deficits. 

## Discussion

Our team reports the successful salvage of both mother and infant despite an unexpected occurrence of MCA. Her only risk factor was advanced maternal age. She had no preexisting cardiac disease, no known ROM, trauma, or hemorrhage which are usually found in MCA [2]. She was in early labor and experienced complete cardiovascular collapse over the course of 1-2 minutes. 

After delivery, we did not achieve immediate ROSC. We continued resuscitation, noting some electrical complexes which may have been agonal or an extremely bradycardic pulseless electrical activity (PEA). After ROSC, there was postpartum hemorrhage and development of DIC which is known to occur in 89% of PMCDs [7]. This was particularly challenging in the face of right heart failure from PE and stunned myocardium from prolonged CPR. In addition, in the setting of a PE, uterotonics such as methergine should be avoided as it causes pulmonary and coronary vasoconstriction.

The successful outcome of this case was facilitated by the prearrangement of an obstetric rescue team based on SOAP guidelines [8]. This provided an immediate response by anesthesiology, obstetrics, nursing, pharmacy, and respiratory therapy. Within three minutes, there were four anesthesiologists, two obstetricians, and one intensivist as well as roughly 15 nurses, mid-levels, pharmacists, and technicians. A prechecked bedside caesarean tray was utilized as opposed to going to the operating room which increases PMCD survival to 72% from 36% [1].

The largest United States study of MCA including 56,900,512 deliveries found that AFE accounted for 13% of MCAs while PE accounted for 7% of arrests [3]. Another large study of 94 reported cases of MCA found 20% due to trauma, 19% from cardiac disease, 18% from severe preeclampsia, 13% from AFE, and none from a venous embolism [5]. One study group that did find a high incidence of PE used the Netherlands Obstetric Surveillance system from 2013-2016 and found that 24% of MCAs were due to a thrombotic PE (18% hemorrhage, 16% AFE, 42% other causes) [3]. This could be due to an unusually high rate of smokers (35%) [3]. Furthermore, out of nine women who suffered an MCA from a thrombotic PE, only one survived (88.9% mortality) [3].

In retrospect, we know this arrest was due to a PE, but there is insufficient evidence to make a definitive diagnosis of AFE versus thrombotic PE. Her initial presentation was a strong contraction followed by chest pain, shortness of breath, and a cough. Both AFE and thrombotic PE present in this fashion. She also developed DIC, but 89% of PMCDs develop DIC regardless of their etiology. A placental abruption was noted, but there was no ROM, and 92% of women experience ROM prior to AFE [9]. CT confirmed a PE, but an ultrasound of the leg veins was negative. Pregnancy is a pro-thrombotic state [9], and maternal uterine vein thrombosis has been described [10]. A cardiac echocardiogram performed immediately after the case showed suspicion for right ventricular thrombus and McConnell’s sign, but this thrombus was not confirmed in subsequent studies; McConnell’s sign could be due to significant pulmonary vascular resistance from an AFE.

## Conclusions

Healthy, full-term parturients may experience a sudden MCA during labor in the absence of pre-existing cardiac conditions or ROM. AFE, through an unknown placental abruption or thrombotic PE triggered by dislodgment of thrombus in the uterine or deep pelvic veins, may lead to MCA. Due to the size of the full-term uterus and fetus, this event necessitated PMCD. This extremely rare event can be successfully managed if there are pre-existing teams and protocols established and practiced in advance. 
